# Clinical Decision Support Tool for Early Pancreatic Cancer Detection in Primary Care: Simulation Study

**DOI:** 10.2196/79209

**Published:** 2026-02-06

**Authors:** Javiera Martinez-Gutierrez, Kaleswari Somasundaram, Christina Maresch Bernardes, Meena Rafiq, Silja Schrader, Susan Jordan, Sophie Chima, Lucas De Mendonca, Kit Huckvale, Barbara Hunter, Jo-Anne Manski-Nankervis, James Lawson, Katrina Anderson, Vivienne Milch, Rachel E Neale, Jon Emery

**Affiliations:** 1 Department of General Practice Medicine, Dentristry and Health Services The University of Melbourne Melbourne Australia; 2 Collaborative Centre for Genomic Cancer Medicine The University of Melbourne Melbourne Australia; 3 Department of Family Medicine Faculty of Medicine Pontificia Universidad Católica de Chile Santiago Chile; 4 School of Public Health The University of Queensland Brisbane Australia; 5 Population Health Program QIMR Berghofer Medical Research Institute Brisbane Australia; 6 Data Connect Victorian Comprehensive Cancer Centre Melbourne Australia; 7 Department of Behavioural Science and Health University College London London United Kingdom; 8 The Digital Health Validitron Centre for Digital Transformation of Health The University of Melbourne Melbourne Australia; 9 Department of Primary Care and Family Medicine Lee Kong Chian School of Medicine Nanyang Technological University Singapore Singapore; 10 National Health Group Polyclinic National Healthcare Group Singapore Singapore; 11 Cancer Australia Sydney Australia; 12 Caring Futures Institute Flinders University Adelaide Australia

**Keywords:** digital health, general practice, simulation, clinical decision support, early detection, cancer

## Abstract

**Background:**

Early detection in primary care could improve pancreatic cancer survival, but diagnosis is often delayed due to the low prevalence of the disease, the nonspecific nature of early symptoms, and the broad range of conditions and volume of consultations managed by general practitioners (GPs). In Australia, improving pancreatic cancer outcomes, including via earlier diagnosis, is a priority being progressed under the National Pancreatic Cancer Roadmap developed by Cancer Australia. Computerized clinical decision support systems (CDSSs) have shown promise in aiding timely cancer diagnosis; however, barriers to adopting CDSS such as mistrust of the recommendations or not being embedded in the clinical workflow remain. Simulation techniques, which offer flexible and cost-effective ways to evaluate digital health interventions, can be used to test CDSS before real-world implementation.

**Objective:**

This study aims to assess the acceptability and feasibility of identifying patients with symptoms associated with pancreatic cancer through a CDSS within a simulated environment.

**Methods:**

We developed a CDSS that interacted with an electronic health record used in general practice to identify patients with symptoms, which may indicate pancreatic cancer (unintended weight loss or new-onset diabetes), in a simulation laboratory for digital interventions. We tested it by inviting GPs (n=11) to use the CDSS, with patient actors simulating specific clinical scenarios. We then interviewed GPs about the interaction to assess the acceptability and feasibility of the CDSS in their clinical practice. We used thematic analysis and 2 relevant frameworks to analyze the data.

**Results:**

GPs found the CDSS easy to use, unobstructive, and effective as a prompt to consider investigations for people with risk factors for pancreatic cancer. However, they expressed concerns about possible overtesting, financial costs, and the potential for anxiety in patients with a very low probability of having cancer.

**Conclusions:**

While GPs found the tool useful and compatible with their workflow, concerns about overtesting, lack of evidence, and cost-effectiveness were identified as barriers. GPs favored a stepwise approach to investigations rather than immediate imaging. Despite the overall acceptability of the tool, additional evidence to underpin clinical recommendations is necessary before implementing a CDSS with these specific recommendations for pancreatic cancer in primary care.

## Introduction

According to the World Health Organization, cancer was responsible for 10 million deaths in 2020 [1]. This striking figure underscores the magnitude of the global health burden created by the disease. Early cancer detection has the potential to improve patient survival and quality of life. In addition, timely diagnosis can reduce the need for aggressive treatments and lessen the overall burden on health care systems [2-4].

The World Health Organization recommends screening for cervical, breast, and colorectal cancers [5,6] with lung cancer screening being introduced in some high-income countries [7]. There are also some country-specific screening programs for cancers where the incidence is high (eg, screening for gastric cancer in Japan) [8]. For most other types of cancer, detection relies on patients presenting with symptoms in the health care system [9].

Most patients with cancer with symptoms first consult in primary care, and delays in diagnosis may occur due to the nonspecific nature of these symptoms, the high number of conditions managed, and the low incidence of cancer in primary care [10,11]. Clinical guidelines provide information about investigations in patients with symptoms suggestive of cancer [12]; however, they are not always adhered to [13] and timely access to this information at the point of care may support their implementation [14].

Clinical decision support systems (CDSSs) can be an efficient way of bringing evidence-based information to practice and facilitating cancer diagnosis. A recent systematic review of CDSS designed to improve cancer diagnosis in primary care showed their potential to optimize the quality of cancer referrals and reduce time to diagnosis. However, there are multiple barriers to implementation, such as poor integration into the practice workflow, lack of general practitioner (GP) time to consider the information, and distrust in the recommendations [15,16].

CDSSs have been developed in the United Kingdom, using symptoms and test results to calculate the likelihood of undiagnosed pancreatic cancer [17-19]. However, these tools face significant challenges:

1. Limited access and adoption: Research indicates that only 36% of clinics have access to these tools, with usage rates even lower at 16%. Time constraints and lack of awareness in clinical settings are the primary reasons cited for this low adoption [20].
2. Lack of specific follow-up recommendations: These tools do not provide standardized guidance at point of care on how to proceed based on the risk assessment results. Consequently, GPs are left to determine the appropriate course of action when presented with a patient’s calculated risk of pancreatic cancer, without specific recommendations for follow-up care.

Clinical decision support tools are being developed in Australia to incorporate current guidelines. However, they face similar challenges in implementation and adoption, highlighting the need for user-centered design and robust evaluation to ensure their effectiveness in real-world clinical settings [21-23].

Pancreatic cancer, although relatively infrequent, is the sixth most common cause of cancer death worldwide, accounting for more than 460,000 deaths in 2022 [24]. Approximately 80% of pancreatic cancer cases are diagnosed when they are not amenable to surgical resection, and this is the primary driver of the poor survival [25]. In Australia, pancreatic cancer has become a health priority, as it was the third most common cause of cancer death in 2024 [26].

The National Pancreatic Cancer Roadmap was developed by Cancer Australia to reduce the burden of pancreatic cancer in a range of ways, including improving primary health care professional recognition of signs and symptoms for pancreatic cancer [27]. As part of this initiative, a working group was established by The University of Queensland in collaboration with The University of Melbourne and Cancer Australia to identify the need for, and characteristics of, a potential CDSS for pancreatic cancer in primary care.

The working group included clinical professionals (medical specialists including GPs, gastroenterologists, hepatobiliary surgeons, endocrinologists, and oncologists), public health specialists, epidemiologists, cancer policy experts, and consumer representatives. Acknowledging the limitations of existing literature regarding predictive value of unspecific symptoms, the working group agreed that “unintended weight loss” and “new-onset diabetes” could be used as flags to potentially alert GPs about the possibility of pancreatic cancer. The choice of these 2 flags and their recommendations was based on evidence of their association with pancreatic cancer.

Studies have shown that patients with pancreatic cancer are approximately 8 times more likely to have been diagnosed with diabetes within the past 12 months [28], and pancreatic cancer is the underlying cause of new-onset diabetes in up to 1% of patients aged 50 years [29,30]. Failure to respond to initial antidiabetic therapy is a potential indicator of pancreatic cancer as the cause of the diabetes [23]. These associations are not commonly recognized by GPs, likely because diabetes occurs commonly, and pancreatic cancer is rare and not usually highlighted in diabetes guidelines for general practice [31].

Unintended weight loss is a red flag for cancer, but it can be caused by most cancer types and is commonly attributed to other underlying conditions [32-34]. A systematic review showed that primary care patients with unintended weight loss were 12.5 times more likely to be diagnosed with pancreatic cancer than control patients [35]. Providing information to GPs about when to prioritize investigations of the pancreas in patients with unintended weight loss was thought to be of value [16].

One way to identify and potentially address barriers related to acceptability and usability before implementation of such a CDSS, is to test it in a simulated environment [36]. We chose a simulation approach to identify barriers related to the acceptability and usability of the CDSS before its implementation. Simulation in this particular case offered several advantages [37,38]:

1. Addressing rarity of symptoms: Unintended weight loss, an indicator for pancreatic cancer, is rare in primary care (1% prevalence). A randomized controlled trial would require substantial time and resources; simulations enable efficient investigation of these infrequent events without waiting for real-world occurrences.
2. Ethical considerations and evidence gap: Due to the nonspecific symptoms of pancreatic cancer, there is little consensus on the evidence supporting a trial for the CDSS. Simulations provide a safe alternative for testing without prematurely implementing the solution in clinical settings.
3. Flexibility and efficiency: Simulation allows for rapid testing of various scenarios, making it more cost-effective and quicker than large-scale clinical trial [39]. This method identifies potential issues early and informs necessary improvements, ultimately bridging the gap between development and real-world evaluation of digital health solutions.

We aimed to conduct an initial investigation of the potential use of including 2 flags into a CDSS for pancreatic cancer detection in a simulated environment: unintended weight loss and new-onset diabetes in the GP electronic health record (EHR).

The insights gained from the simulation will provide essential guidance for the subsequent empirical phase. These findings will help refine the study’s methodology and strengthen its overall design. Consequently, the transition to real-world application can be more seamless and effective.

## Methods

### Study Design

**Figure 1:**
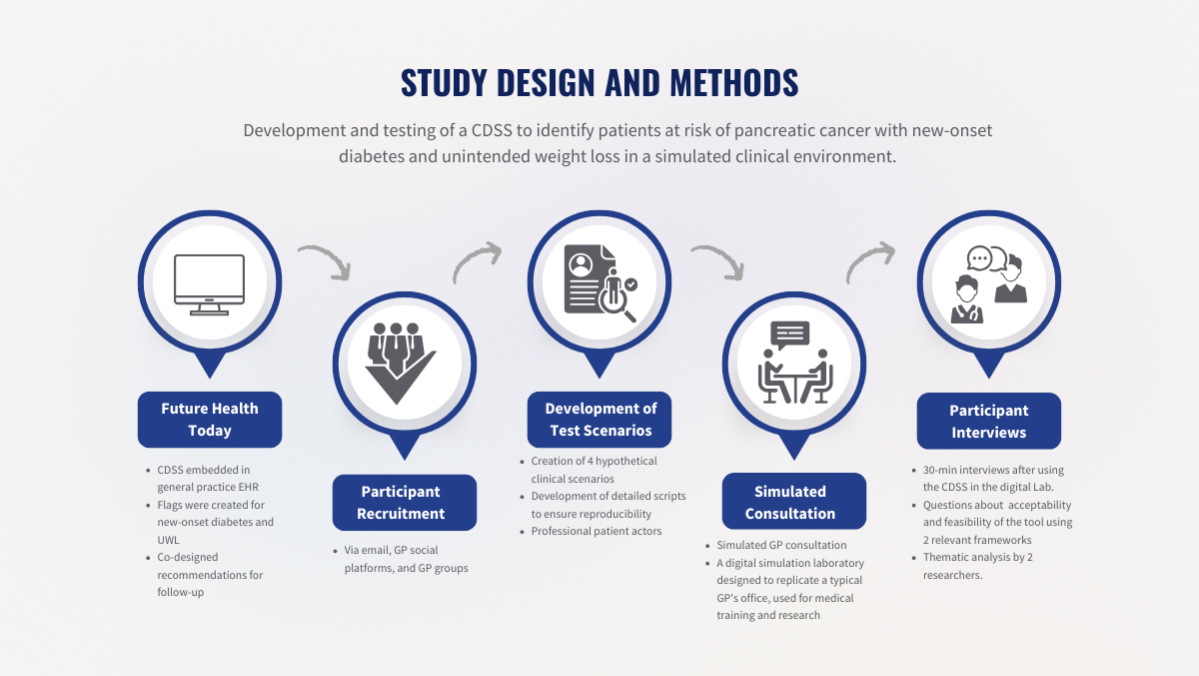
Study design and methods. CDSS: clinical decision support systems; GP: general practitioner; UWL: unintentional weight loss.

We conducted a simulation study of a CDSS embedded in general practice EHR to identify patients with new-onset diabetes and unexplained weight loss at risk of cancer, where GPs trialed the tool in simulated consultations and were interviewed about their perceptions. An overview of the process is depicted in Figure 1.

We describe the process and each of their components below.

### CDSS: Future Health Today

We used the Future Health Today (FHT) software (University of Melbourne) [40], a CDSS designed by the Department of General Practice and Primary Care at The University of Melbourne to assist general practices in identifying and managing health conditions. FHT was co-designed with clinicians working in general practice and consumers; it is embedded into a practice’s EHR and offers recommendations at the point of care [41]. It also includes links to complete relevant clinical guidelines and resources. This system is an active, asynchronous CDSS. Active means it proactively generates recommendations based on EHR data without requiring the GP to input specific information. Asynchronous indicates that the algorithms run once daily, analyzing newly available data. However, because the information is not processed in real time during a patient visit, the recommendations appear only the next time the GP accesses the EHR.

**Figure 2:**
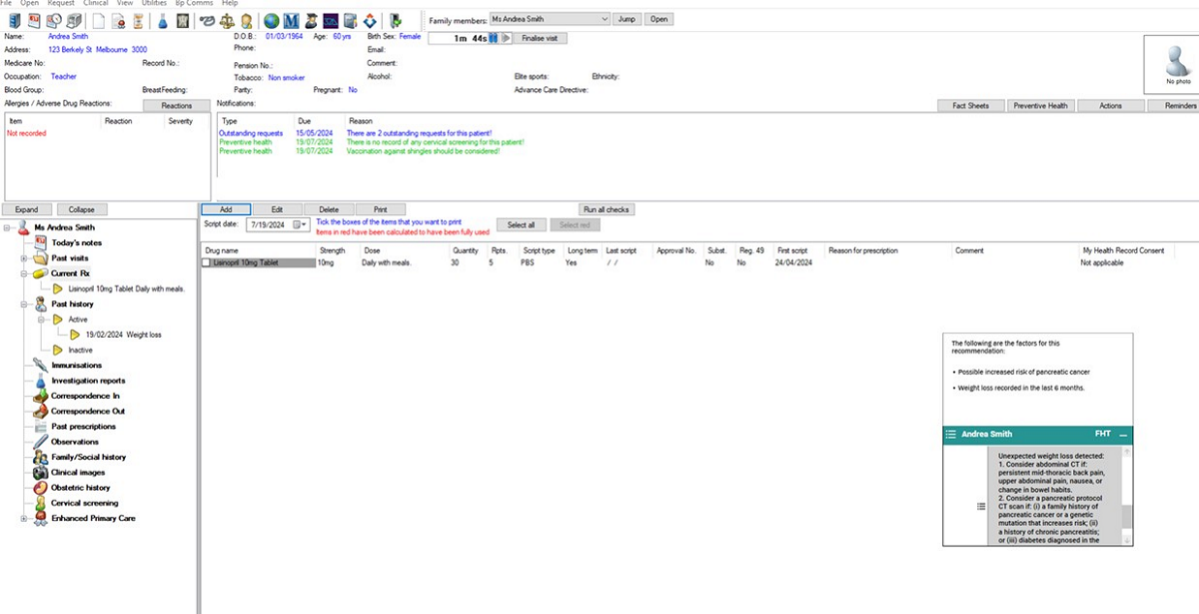
Recommendations for a patient with unexpected weight loss, as displayed in the electronic medical record.

We embedded flags for unintended weight loss and new-onset diabetes with recommendation for follow-up and included links to the clinical guidance developed by the working group. A snapshot of the pancreatic cancer recommendations, as they were displayed in the EHR, can be seen in Figure 2.

The alert message with recommendations was displayed at the bottom right-hand corner of the screen in the EHR. This alert pops up as the clinician opens the EHR of a patient with unexpected weight loss. GPs could hover over or click on the screen to get information about the reasons for the alert and access resources (the information displayed here does not belong to a real patient). Example of wording:

Unexpected weight loss detected:

1. In women aged 60-79 years, prioritize testing for thyroid function and screening for depression while considering cancer investigation.
2. Consider abdominal computed tomography (CT) if persistent midthoracic back pain, upper abdominal pain, nausea, or change in bowel habits.
3. Consider pancreatic protocol CT if (1) a family history of pancreatic cancer or a genetic mutation that increases risk, (2) a history of chronic pancreatitis, or (3) diabetes diagnosed in the previous 6 months.

The recommendations are described in the subsequent sections.

### Unintended Weight Loss

For patients with unintended weight loss, GPs were advised to consider common differential diagnoses based on age and sex [42], and consider referring patients for an abdominal or pancreatic protocol CT if additional risk factors were present (ie, persistent midthoracic back pain, upper abdominal pain, nausea, or change in bowel habits).

### New-Onset Diabetes

For patients with new-onset diabetes, two categories of advice were provided: (1) *Urgent investigation*: For patients with specific risk factors or symptoms (chronic pancreatitis, family history of pancreatic cancer, or who have had persistent midthoracic back pain or upper abdominal pain), GPs were advised to consider urgent CT using a pancreatic protocol. (2) *Nonurgent investigation*: In patients with new-onset diabetes but no other symptoms or risk factors, GPs were advised to retest the hemoglobin A_1c_ (HbA_1c_) 3 months after commencing medication and, if the HbA_1c_ had not responded in accordance with expectations and no cause could be identified (eg, medication noncompliance), to consider a pancreatic protocol CT. These recommendations were developed using GP and expert input, current guidelines [22], and best evidence available at the time [21,23,28,30,35,42-44].

### Recruitment

We emailed a convenience sample of 72 GPs from a database available at the Department of General Practice, focusing on those with current connections to the department, as they needed to attend the simulation laboratory, and posted on social media platforms and groups associated with the University. We excluded from our invitation GPs who had participated in similar studies to avoid potential bias from their acquired “expertise” in simulated scenarios. We recruited all GPs who responded to our initial recruitment invitation, distributed via email and social media groups, and were able to attend the simulation laboratory.

### Simulation Experience

The “Digital Health Validitron” at The University of Melbourne, hosted by the Centre for Digital Transformation of Health, comprises a simulation laboratory for designing, developing, validating, and evaluating digital health solutions [45,46]. The laboratory includes a physical recreation of a general practice clinic and a virtual “sandbox” (a virtual machine used to run software in a testing environment) hosting an EHR that, together, allowed our CDSS to be tested in a controlled environment.

Four hypothetical patient scenarios were created (2 for unintended weight loss, and 2 for new-onset diabetes; 1 female and 1 male patient for each condition). The cases were designed to reflect varied clinical scenarios. New-onset diabetes was included as relevant background in the EHR and simulated patient’s history, with patients presenting with nonspecific symptoms such as fatigue and abdominal pain as their main reason for visit. For unintended weight loss, in 1 case it was included as background information from a previous visit, while in the other, it was the main presenting symptom. The cases were developed with input from GPs in our advisory group to ensure real-world fidelity and each script provided a detailed description of the case including behavioral cues (eg, patient looks fatigued; do not provide this information unless asked, etc). The scenarios were played by professional simulated patient actors from the Department of Medical Education at The University of Melbourne (Multimedia Appendix 1).

We invited GPs to test the scenarios in a simulated consultation, with CDSS recommendations appearing in the EHR. We informed participating GPs that they were testing a CDSS designed to identify and assist with the investigation of nonspecific symptoms in primary care. They were not made aware that the study focused specifically on pancreatic cancer. A plain language statement describing the study was provided to all participants and GPs and patient actors–signed consent forms were collected prior to the simulation session. Each session took 10-15 minutes, consistent with consultation times in Australian general practice, and was observed, filmed, and audio recorded by the researchers through a one-way mirror. Before the simulation session, the GPs were introduced to the CDSS and given the option to review the patients’ EHR prior to the consultation, mirroring usual practice in primary care. Each GP was presented with 2 patient scenarios, determined by the gender of the available patient actor at the time of the session.

### Interviews

**Figure 3:**
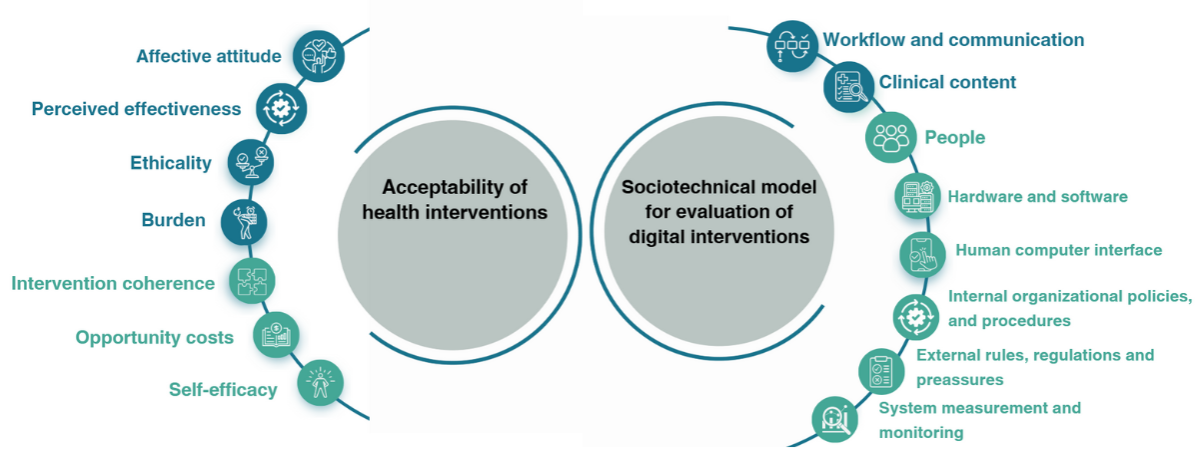
Frameworks used and their dimensions (adapted from Sittig and Singh [47,48] and Sekhon et al [49]).

Following completion of the simulated consultations, the GPs participated in a single semistructured interview on site (Multimedia Appendix 2), assessing the acceptability, feasibility of the CDSS, and its impact on workflow. There were no external people to the study present during the simulation or the interviews. The interview guides were developed using relevant dimensions of two published frameworks: (1) sociotechnical model for evaluation of digital interventions by Sittig and Singh [47,48], and (2) the acceptability of health care interventions by Sekhon et al [49]. A depiction of the 2 frameworks and their dimensions can be found in Figure 3 [47-49].

We selected these 2 frameworks over other well-established models as our research required a broad scope that encompassed the technical aspects and the wider contextual and human factors involved in implementing a CDSS in primary care. These frameworks also not only focus on general technology use and acceptance but, particularly important, they are health care specific. Both chosen frameworks are relatively recent developments in the field, which incorporate learnings from earlier models while addressing some of their previous limitations. The Theoretical Framework of Acceptability by Sekhon et al [49] uses a multidimensional approach to assess the acceptability of health interventions in general. Its 7 dimensions—affective attitude, burden, ethicality, intervention coherence, opportunity costs, perceived effectiveness, and self-efficacy—offer a rich understanding of how interventions are perceived from the user’s perspective.

In contrast, the sociotechnical model is specifically tailored for evaluating digital interventions. It provides a comprehensive lens that incorporates both technical aspects (eg, hardware, software infrastructure, and human-computer interface) and user, procedural, and contextual dimensions (eg, people, workflow and communication, internal organizational policies, procedures, and culture).

By using these 2 frameworks together, we were able to comprehensively explore and identify a wide range of barriers and facilitators to implementing and testing the CDSS in a safe and controlled environment. A description of each category can be found in Table S1 in Multimedia Appendix 3). Interviews lasted approximately 30-45 minutes.

### Data Analysis

The Sittig and Singh [47,48] and Sekhon et al [49] frameworks were systematically integrated into our thematic analysis process as follows:

1. *Framework integration*: Each dimension from both frameworks was used as an overarching theme in our codebook. This approach ensured a comprehensive and structured analysis aligned with established theoretical concepts.
2. *Coding process*: JMG and KS independently coded the transcribed interviews using NVivo (version 14; Lumivero). We systematically categorized each code under the relevant themes derived from our theoretical frameworks, ensuring a consistent application of the frameworks’ components.
3. *Interrater reliability*: To maintain interrater reliability, both coders adhered strictly to the descriptions of the framework dimensions provided in Table S1 in Multimedia Appendix 3. We discussed meaning and interpretations of codes through the process. This standardized approach ensured consistency in interpretation and application of the frameworks across coders.
4. *Consensus building*: Any disagreements in coding or categorization were resolved through discussion between JMG and KS until consensus was reached. This process further enhanced the reliability and validity of our analysis.
5. *Synthesis and quote selection*: Following the initial coding, we conducted a comprehensive analysis of the categorized codes, synthesizing findings within each theme. Representative quotes were carefully selected to illustrate key concepts and insights from each category, ensuring a clear link between raw data and our theoretical frameworks.

We used reflexive thematic analysis, which conceptualizes meaning as constructed through the researcher’s interpretative process rather than inherent in the data. This approach recognizes that new meanings are always theoretically possible, as analysis is a situated, reflexive, and theoretically embedded practice of knowledge generation. Consequently, the concept of data saturation, which assumes a point where no new information emerges, was not suited to our study. Instead, our sampling strategy focused on recruiting participants who could provide rich, relevant information to address our research questions. This approach ensured that we captured a range of perspectives from those willing and able to participate, rather than aiming for a predetermined point of “informational redundancy” [50].

Interview participants were not asked to provide feedback on the transcripts, nor the findings and no repeat interviews were conducted. Field notes were not developed during or after the interviews. We followed the COREQ (Consolidated Criteria for Reporting Qualitative Research) checklist (Multimedia Appendix 4) to present our findings [51].

### Researcher Characteristics

JMG was the lead investigator for this study and was assisted by KS. JMG is a trained GP and PhD candidate at the Department of General Practice and Primary Care, The University of Melbourne. KS, a researcher in the Department, holds an MPhil, and has been actively involved in primary care research since completing postgraduate studies. They are both females, experienced in qualitative research, and together they bring a complementary blend of clinical expertise and research experience to the study. Neither had an established relationship with study participants.

### Ethical Considerations

Ethics approval for this study was obtained from The University of Melbourne Human Research Ethics Committee LNR 4D (approval ID: 2024-29048-51715-3). All participants provided written informed consent prior to or on the day of the simulation and were reminded that they could withdraw at any time. Audio recordings and transcripts were stored securely on an institutional, password-protected server, with all personal identifiers removed to ensure confidentiality. GPs received reimbursement for their participation (Aus $150 [approximately US $98]), and actors were compensated according to The University of Melbourne’s standard pay scale. No other information was provided regarding personal goals or reasons for doing the research.

## Results

### Overview

Of the 72 GPs invited, 12 responded and 11 participated (1 GP was not available at the time of the simulation sessions). Approximately half of the GPs encountered female patient scenarios. The demographic characteristics of the participants are described in Table 1. Seven GPs were aged in their thirties and had fewer than 10 years in practice, 8 were female, and 7 were Australian born. Most GPs practiced in metropolitan practices, 1 had experience in rural practices, and 2 worked in an Aboriginal Medical Service.

We reported relevant themes based on 2 key criteria: the volume of information obtained, and their assessed relevance for implementation, as determined by our research team. While we acknowledge that themes may be interrelated across frameworks, we made a deliberate choice to report them separately for the sake of clarity and to avoid overcomplicating distinct aspects of our analysis.

**Table 1:** Demographic characteristics of general practitioner participants (N=11).

GP^a^ characteristics		Participants, n
**Age (years)**		
	30-39	7
	40-49	2
	50+	2
**Sex**		
	Male	3
	Female	8
**Years of experience**		
	1-10	7
	11-20	2
	21-30	2
**Practice setting**		
	Urban	8
	Regional	1
	Aboriginal Medical Service	2
**Country of birth**		
	Australia	7
	Overseas	4

^a^GP: general practitioner.

### Theoretical Framework of Acceptability

The key aspects of the framework related to “affective attitude” toward the flag, its “perceived effectiveness,” its “burden,” and any “ethical concerns” related to implementing the recommendations. Feedback on the intervention’s acceptability highlighted GPs’ “self-efficacy,” reflecting their confidence in using the tool to benefit both their practice and their patients.

### Affective Attitude, Perceived Effectiveness, and Ethicality

Overall, participants appreciated the recommendations, viewing them as helpful reminders and safety nets to ensure that they did not overlook important aspects of patient care (regardless of the condition with which a patient presents) in typically busy general practice. In general, GPs with fewer years in practice thought that a CDSS like this would be very effective at identifying patients at risk of cancer, given their own lack of experience diagnosing patients with cancer, whereas more experienced GPs, while liking the idea of a reminder, thought that they would trust their clinical experience and would not radically change their approach.

So, it's an interesting tool. But it'll just augment your thinking, and just remind you of things, which is always useful when you're busy and tired.

GP9

Collectively, the GPs valued the gentle language of the recommendations and resources provided by the CDSS for further reading.

I normally hate flow charts..., But this is pretty straightforward.

GP2

While they collectively agreed that the point-of-care recommendations served as gentle reminders, some GPs also expressed concerns that it might prompt them to focus solely on pancreatic cancer, potentially overlooking other possible diagnoses, for patients with unintended weight loss and new-onset diabetes.

But it did kind of push me towards focusing on that. ...And even though I guess it's really important to know about pancreatic cancer, would it make me forget about other diagnoses.

GP2

Some GPs highlighted the possibility that the flags would increase the risk of overdiagnosis and lead to too many computed tomographic scans being ordered.

Yeah, if I take it literally, I’d be doing CT scans on a huge number of people.

GP4

Half of the GPs were concerned that recommendation to do CT might influence junior GPs to follow point-of-care recommendations without considering the patient history as a whole, due to the medicolegal risk if they did not engage with the recommendations.

I think if you are prompted to look for a condition and then you personalise the decision and don’t look for the condition, and it turns out, the patient does have the condition you’re potentially legally in a bit of a pickle.

GP1

### Self-Efficacy

More than half of the GPs expressed their confidence using the resources provided by the recommendations to enhance their knowledge regarding pancreatic cancer and at the same time confidently communicate to their patients that their suggestions for further testing are based on recent evidence.

That's good. So, if it does that, then I think in the long run, it would improve my safety, it would teach me a few things like the relationship between diabetes and pancreatic cancer, which I think is not in as much in my mind as it should be. So, it’s a good one to be setting as an example, I’m sure that's why you chose it.

GP2

### Burden

Although the use of the tool did not represent a burden in itself, most GPs highlighted that they would think twice before following the recommendation to consider a CT, given the time and financial burden on their patients.

...I like to have some sort of baseline something before sending people off to potentially expensive and time-consuming tech.

GP4

### Sociotechnical Framework

This framework focuses on factors and processes necessary to implement a digital health intervention. The qualitative data heavily reflected how the clinicians integrated the intervention into their existing workflows and communication strategies, as well as their concerns when discussing recommendations with patients. They also shared their expectations about clinical content to encourage more frequent use of the recommendations.

### Workflow and Communication

This was the second most dominant theme arising from the interview data. Fitting the point-of-care recommendations into the workflow was easy, GPs found it nonintrusive, and almost all preferred looking at the recommendations prior to patients’ arrival for consultation as their routine practice.

I’m someone that looks at the patient’s file before I see them, always. So, I’d already sort of looked at the tool and thought about if it needed, if anything needed to be incorporated. So, then I just did the consult as normal once the patient was there.

GP6

Most GPs reflected that they prioritize communicating effectively to patients about their decision-making and recommending new tests without causing the patient undue alarm. GPs felt that this was a particular concern for patients with new-onset diabetes, resulting in a potential communication barrier.

Yeah, it's hard...because...you want the patient to know that it’s important...because it could be something serious. But how do you communicate that without making them panic?

GP3

### Clinical Content

Almost all GPs suggested that they would value statistical evidence about the incidence of pancreatic cancer in patients with the symptoms flagged to further guide their decision-making.

But I would love to...(have) the overall incidence or prevalence of pancreatic cancer...what the likelihood of that person actually having pancreatic cancer would be because that would change my decision making.

GP7

Some GPs expressed the importance of HbA_1c_ profiling for new-onset diabetes to better understand whether it was a gradual increase in the reading or sudden spike to be able to justify their clinical decision in suggesting a CT.

I guess it's because...we’re talking about HbA1c that's high enough, rather than a very gentle creep over many years.

GP5

A summary of the major themes and further quotations is shown in Table 2.

A summary of each GP’s opinions and thought processes for the entire simulation, along with their respective quotations, can be found in Table S1 in Multimedia Appendix 5.

Although the overall attitude toward a CDSS for pancreatic cancer was positive, there were important concerns and barriers that would need to be addressed for implementation. A major concern was the risk of overtesting, which not only poses a financial burden but also heightens patient anxiety due to the uncertainty of a potential cancer diagnosis. Textbox 1 provides a summary of the facilitators, barriers, and concerns identified.

**Table 2:** Examples of quotations from main relevant themes.

Relevant themes	Representative quotes
**Framework: Acceptability for Health Interventions [49]**	
Affective attitude	“I like it, I think patients don't tell you what they need. Sometimes, with symptoms you can be, you can become really siloed in which direction you're headed. So being made or being reminded of those rare but not to miss things is useful.” [GP1] “It's still your judgment, isn't it? But it's not I know; it's not trying to replace your clinical job. It's just trying to prompt you to think of things. So, it's saying yeah, consider, I mean, I think that's, that's fair enough. Yeah. There'd be some people who maybe, particularly if there were other risk factors, I guess it would be good to think about. Yeah, I think that's why it's very concise and clear.” [GP3]
Perceived effectiveness	“Yeah, it's a quite a non-intrusive tool, you know, you can look at it there for guidance if you need to. But you can also have sort of your own clinical judgment guiding the consult. And you get better at that, I think at first, it can be distracting and can perhaps derail you, or perhaps make you over investigate or second guess your own approach to a presenting complaint. But I think now that I've used, I've seen and seen and used this a couple of times, you get better at rationalising what you're doing, and knowing where to look for the justification, you know, double checking guidelines if you want to, and then making it work for you.” [GP8]
Ethicality	“But maybe for doctors who are a bit newer in training are more anxious or nervous or maybe new to Australian healthcare system and our learning language and how things work. It may... I think that just need to be trained to not use that as a, ‘this is what you should be doing’ tool. Because I’ve had registrars who have done something like that before they’ve seen something like this and being like I have to screen everyone for pancreatic cancer. That’s the only thing I can think of as a drawback.” [GP9] “But the risk in that is also that you might over call things and you don't know where to stop. And I certainly have colleagues who can over investigate everything and refer everything to specialists just because there's a minor abnormality.” [GP11]
Self-efficacy	“I mean, they’re gentle in their language, because it says consider. You know what I mean, it still sort of leaves it with you.” [GP8] “I would read over this, the first time I've come across this because this is new information to me. Not everyone would, particularly because of the length of it (resource document). And I think it depends on your patient, I think the newer age patients are often coming to you with some information themselves. So, they often or particularly stronger use around investigation. So, you, I think you need to be better informed yourself. So, this is where like for a patient who, who, you know, was particularly hesitant to go and have screening, they might be asking me, well, can you give me more information? And so, I pull up that guideline and say, well, someone's actually looked into this, and that evidence shows is that, you know, there's some increased risk with these issues and lesser risk with these issues.” [GP1]
Burden	“But again, if this guideline said any unexplained bloating, goes straight to pelvic CT, I probably would follow what the future health today told me as long as the patient was happy with it. And I guess you will say you making all these other decisions aren't cheap.” [GP2]
**Framework: Sociotechnical for digital interventions [48]**	
Communication and workflow	“Simply because if you were just asking risk factors, and then she didn't have any, that might be okay. But then she comes up with another risk factor, then you actually have to address that, and then you have to deal and because you've said the word cancer, then you usually have to deal with the fact that they've heard cancer and then are stressed about it.” [GP7]
Clinical content	“I would probably like...the probabilities so that...I would be less worried if I knew it's just,...the risk goes from 1% to 2%. That's a completely different conversation than if the risk goes from 1% to 50%, isn't it?” [GP2] “I really think it is because I think if you have doctors that are like, I'm quite confused by this, and I want to learn more, or I'm quite surprised by these recommendations. I want to learn more. It's good that you direct them to what you want them to look at. Or where they can find more. That's a good resource.” [GP5] “I think consider a CT scan leads me to where I was before, which is that I wouldn't order, this is a very soft recommendation. So I guess again, that...statistics or something to justify. And, I mean, I like numbers. So, weight loss of X percent or X amount.” [GP6]

**Key facilitators, barriers, and concerns identified.**

**Facilitators**

- Resources provided through point of care, especially the flowcharts.
- Gentle language of the recommendation gives flexibility to clinical discretion.
- Seamless fit into workflow via point of care.
- Useful as safety net and reminder in a busy general practice.

**Barriers**

- Lack of statistical evidence on pancreatic cancer risk in patients with these presentations.
- Need for hemoglobin A_1c_ profiling for patients with new-onset diabetes.
- Longer consultation time to action the recommendation.
- Several consultations needed to gradually introduce the recommendation for patients with new-onset diabetes.
- Time and cost of computed tomography.
- Perception of potential alarm in patients if the topic of cancer is addressed.
- Potential medicolegal risk of not actioning or actioning the recommendations.
- Concerns
- Potential bias in targeted diagnosis and fear of missing other conditions.
- Overtesting and reduced threshold for computed tomography in patients.

## Discussion

### Principal Findings

This simulation study aimed to assess the acceptability and feasibility of a CDSS to prompt GPs to consider investigations for potential pancreatic cancer in people with unintended weight loss or new-onset diabetes. Testing the CDSS in a simulated environment was valuable, revealing the potential benefits while highlighting barriers to implementing the recommendations in real-life settings.

Most patients with cancer have multiple GP consultations before diagnosis. A Victorian study found that 34% of Australian patients with cancer had 3 or more GP visits before being referred to a specialist. The delays varied by cancer type; for instance, patients with pancreatic cancer or myeloma, who typically present with nonspecific symptoms such as abdominal or back pain, were more likely to have had multiple GP visits than those with breast cancer or melanoma [9]. This highlights the need for tools to aid early cancer detection, in particular, for cancers, including pancreatic cancer, that are rare and frequently present with nonspecific symptoms. In line with international literature on CDSS for early cancer detection [16], our study identified both facilitators and barriers to implementing a pancreatic cancer CDSS. Our CDSS was acceptable and unobstructive and provided easy-to-read resources for GPs. A systematic review examined studies that used CDSS for skin, colorectal, and other gastrointestinal cancers (not pancreatic). It identified three key barriers to implementation: (1) Mistrust in the tool: this stemmed from ambiguity in the underlying guidelines or discrepancies between the tool’s recommendations and clinicians’ own assessments. (2) The GP’s role as a gatekeeper: GPs were concerned about overreferral, fearing that the tool could overload health care systems with unnecessary referrals due to the low prevalence of cancer in primary care. (3) Impact on workflow: GPs highlighted the potential burden these systems could impose, further straining their already busy schedules [16]. Our results were concordant with these observations, reinforcing the international relevance of these challenges. Specifically, regarding mistrust, years of clinical experience emerged as the sole participant characteristic that notably influenced opinions, particularly regarding both system usability and trust. We observed that junior GPs generally demonstrated greater trust on CDSS and were more willing to accept and follow its recommendations. In contrast, their more experienced colleagues tended to prefer relying on their own clinical acumen.

Our analysis did not reveal any distinct differences in opinions when considering other participant characteristics such as age or gender. This finding suggests that while experience level may play a significant role in CDSS adoption and utilization, other demographic factors had less impact on participants’ views and experiences in our study. It is important to note that given our relatively small sample size, which is appropriate for an in-depth qualitative study, we are cautious about drawing definitive conclusions based on demographic subgroups. The observed differences related to clinical experience provide valuable insights, but further research with larger, more diverse samples would be necessary to confirm and expand upon these findings.

The gatekeeper role was similarly reflected in our findings, with GPs voicing apprehension about the potential for increased referrals and the associated strain on specialist services. Moreover, GPs also highlighted the impact of time constraints during regular consultations, making it challenging to talk about a very unlikely potential cancer diagnosis. This reluctance aligns with global findings [52], emphasizing the universal nature of these implementation barriers across different health care systems and geographical contexts.

However, recommendations based on unintended weight loss were more readily accepted than the recommendation for new-onset diabetes, although there was some concern about the focus on pancreatic cancer that would cause other conditions to be missed. Although most GPs felt that they would follow up patients with unintended weight loss even without the recommendation, previous research shows that unintended weight loss can be challenging to identify in primary care settings. A study conducted in a retrospective cohort in the United States found that primary care clinicians identified only 21% of all cases of unintended weight loss in a 2-year period [53], so a technological solution to both flag weight loss and then prompt GPs to consider cancer (including pancreatic cancer) may be needed.

Despite growing evidence linking new-onset diabetes and pancreatic cancer [29,44], GPs were hesitant to follow recommendations for early CT in patients with newly diagnosed diabetes. There was an overarching concern about the potential for overreferral and overtesting, with associated costs and the potential for harm. Additional barriers included insufficient evidence or knowledge about the association and practical limitations such as cost and availability of CT in rural or remote areas [16,52].

GPs also expressed the need for more information on how to order a pancreatic protocol CT, specific Medicare coverage (Australia’s universal health insurance scheme that provides reimbursement for some investigations), and evidence regarding the incidence of pancreatic cancer among people with new-onset diabetes, as well as the cost-effectiveness of widespread case-finding approaches. Some of this information could be provided through education resources, but there is currently little evidence about whether systematically investigating people with new-onset diabetes with CT would have net benefit, or the costs of this activity. A recent systematic review published in 2025 found that pancreatic cancer screening in high‑risk populations can be cost‑effective. High‑risk populations were defined as those with a lifetime risk greater than 5%, including individuals with a family history of pancreatic cancer, relevant genetic mutations, or new‑onset diabetes within the past 3 years. The review analyzed 10 studies: 6 from the United States, 2 from Japan, 1 from Denmark, and 1 from Sweden. Three studies focused specifically on patients with new‑onset diabetes. Two of these stratified patients aged 50 years and older into high‑ or low‑risk groups based on factors such as age, sex, and abnormal test results; in one, high‑risk patients underwent contrast CT. The third study assessed the cost‑effectiveness of microRNA compared with CA 19‑9, various imaging tests, and no screening in patients with diabetes. Across all studies, the proposed screening strategies were effective at varying thresholds of risk [54]. These findings highlight the need to carefully analyze and communicate risk information to GPs in a way that reduces barriers to implementation. GPs also mentioned the challenges of discussing an unlikely cancer diagnosis and the unnecessary anxiety this may cause to patients. Digital tools can be leveraged to enhance clinician-patient interactions, and we will use this knowledge to inform the design of a feasibility trial currently in process. Research indicates that CDSS can aid communication and shared decision-making when they use non–clinical language and consider individual patient factors [55,56]. Educational material was developed for this purpose as well as focusing on CDSS functionality and coherence, workflow integration, and up-to-date guidelines.

Our study showed that to implement an effective CDSS for pancreatic cancer, it is crucial to first integrate evidence-based resources that highlight the association between pancreatic cancer, symptoms, and new-onset diabetes. It is important that this tool provides comprehensive guidelines on relevant testing, including imaging modalities, to ensure timely and accurate diagnosis. Research has shown that guidelines are more likely to be adopted if they are easy to understand, do not require specific training, are disseminated via multiple channels and have multiple components (reminders, resources, etc), and align with the clinicians’ values [14]. These guidelines should be succinctly and clearly summarized into flowcharts or other visual resources. These features have all been incorporated to the CDSS in accordance with our findings. Incorporating cost-effectiveness analyses can help prioritize testing strategies that maximize patient outcomes while minimizing financial burden. Overtesting and cost have been highlighted by the participants as crucial, and more research is needed to clarify these questions before a CDSS can be widely implemented in general practice. In addition, the tool should offer patient resources, such as educational materials on managing symptoms and accessing support services, to empower patients and enhance their overall care experience. By leveraging these elements, health care providers can make informed decisions, potentially improving early detection rates and optimizing diagnostic pathways for patients with pancreatic cancer.

The Australian Primary Health Care 10‑Year Plan (2022-2032) places digital transformation at the center of a “future‑focused” primary care system, highlighting telehealth, data‑driven insights, and precision medicine as key priorities [57]. CDSSs are central to this vision, enabling more personalized and higher‑quality care. Our CDSS for pancreatic cancer has the potential to support GPs by promoting guideline adherence, standardizing referrals, and prompting timely follow‑up, helping overcome barriers such as limited time and access to specialist resources. As digital‑native generations increasingly expect seamless, technology‑enabled health care, this tool can help demonstrate how digital innovation can strengthen primary care and improve early risk detection.

Current “Optimal Care Guidelines for Pancreatic Cancer” [22] in Australia, while available, lack several key features identified in our study. These guidelines are not readily available at point of care, do not incorporate sufficient visual aids and summaries, and fail to provide easily accessible resources for clinicians and patients. Our CDSS offers evidence-based recommendations aligned with current guidelines while incorporating features specifically requested by GPs. These tailored functionalities can enhance clinical decision-making and streamline workflow in primary care settings

Our research, involving the simulation of a CDSS for pancreatic cancer and collaboration with expert working groups, has informed the development of a new set of guidelines. These guidelines include GP requirements highlighted in this study, offering point-of-care accessibility, comprehensive visual aids and summaries, and resources for both clinicians and patients. This enhanced guideline package has been submitted to Cancer Australia for distribution, aiming to improve early detection rates and optimize diagnostic pathways for patients with pancreatic cancer in alignment with Cancer Australia’s National Pancreatic Cancer Roadmap.

### Strengths and Limitations

We used a simulation approach, which allowed for the controlled testing of a CDSS designed to identify individuals with symptoms that may be indicative of undiagnosed pancreatic cancer. Simulation enabled the exploration of various clinical scenarios, ensuring that the CDSS was evaluated under diverse and realistic conditions without putting actual patients at risk. In addition, it provided valuable insights about barriers and facilitators for the implementation of a tool for early cancer detection, which if successfully implemented, could lead to better patient outcomes and more efficient health care resource utilization [16]. In this setting, simulation effectively fulfilled dual roles. It allowed direct modeling or observation of practical issues relating to usability and workflow. Separately, it provided “priming” to enable practitioners to give rich feedback about the CDSS approach.

Our study has limitations; one being limited geographic diversity. We were able to obtain perceptions toward the tool from male and female participants, junior and senior GPs, and GPs born overseas and in Australia. We could not, however, recruit participants from rural practices. This geographical constraint was unavoidable, as participants needed to attend the simulation laboratory to test the CDSS. Consequently, our findings may not be generalizable to rural or remote health care settings. The metropolitan-only sample potentially introduces bias toward urban health care challenges, resources, and patient demographics, while limiting insights into rural-specific needs and constraints. These factors may impact the applicability of our results to nonmetropolitan environments and hinder the study’s replicability in rural or remote practices. Although evidence-based recommendations for pancreatic cancer follow-up and investigations can support clinical decision-making for GPs in rural and remote areas, challenges such as overtesting, high costs, and limited access to advanced imaging are likely to present significant barriers to implementation in these settings. As with all simulation studies, it is important to note that there is an inherent gap between them and real-world clinical practice. Simulations, while valuable for training and research, may not fully capture the complexities and nuances of actual patient care. These include provider decision-making processes, patient compliance issues, and unforeseen variables in clinical settings. The lack of real diagnostic consequences in simulations could alter the decision-making process of health care providers, potentially leading to choices that may not reflect real-world practices where the stakes are considerably higher. Although we aimed to minimize variability in actor performance by providing detailed scripts, this factor can introduce inconsistencies in the simulated scenarios, potentially skewing results [58]. Actors may not always accurately portray the nuanced symptoms or behaviors of actual patients, which could impact the validity of the simulation. Furthermore, the potential Hawthorne effect, where participants’ behavior may be altered simply by their awareness of being observed, can introduce bias into the simulation results [59]. This effect might lead to artificially improved performance or decision-making that does not accurately represent typical clinical practice, thus potentially overestimating the effectiveness of the simulated intervention.

Our CDSS relied on data recorded in the EHR to identify patients with relevant symptoms and its performance was therefore linked to the quality of data recorded in GP practices. The recommendations were delivered asynchronously, meaning they appeared in the patient’s EHR only at the time of their next appointment. While asynchronous reminders can serve as safety-netting tools by prompting GPs to inquire about specific symptoms during subsequent visits, this approach carries the risk of missed opportunities for real-time investigation. A significant gap may occur between appointments, or the patient may not return at all, potentially delaying critical care. To address this and provide practices with a proactive solution, FHT includes an additional feature not covered in this study. This feature allows health care providers to create lists or cohorts of patients with specific conditions, enabling the clinic to actively recall these patients for follow-up.

Emerging technologies such as artificial intelligence and natural language processing of clinical notes may help identify these patients more effectively and in real time. Furthermore, the outcomes of a simulation may not accurately predict the long-term clinical impact, including how well the tool integrates with existing systems or how health care providers respond to its recommendations in practice.

### Conclusions

We aimed to assess feasibility and acceptability of a CDSS for patients at risk of pancreatic cancer. Our simulation of a CDSS for identifying individuals with unintended weight loss and new-onset diabetes demonstrated that clinicians appreciated the concept of a reminder to consider pancreatic cancer in these patients. However, concerns were raised mainly regarding the potential for overtesting and the associated costs. These findings highlight the need for further research to provide a robust evidence base to support the net benefits of early imaging in patients with potential symptoms of pancreatic cancer.

## Appendix

Multimedia Appendix 1 Patient scenarios.

Multimedia Appendix 2 Interview schedule.

Multimedia Appendix 3 Description of framework dimensions.

Multimedia Appendix 4 COREQ (Consolidated Criteria for Reporting Qualitative Research) checklist.

Multimedia Appendix 5 General practitioner thought process for decision-making.

## Abbreviations

CDSS: clinical decision support systems
COREQ: Consolidated Criteria for Reporting Qualitative Research
CT: computed tomography
EHR: electronic health record
FHT: Future Health Today
GP: general practitioner
HbA1c: hemoglobin A1c

## Data Availability

Unidentified transcriptions and qualitative coded data can be made available upon request secondary to ethics approval. Qualitative codes were developed in an iterative process by the 2 researchers (JMG and SC). They were uploaded to NVivo version 14. They can be shared upon request.
