# Supplementary material for: Clinical Decision Support Tool for Early Pancreatic Cancer Detection in Primary Care: Simulation Study
Source: JMIR Form Res. 2026 Feb 6;10:e79209. doi: 10.2196/79209 (PMC12924040; doi:10.2196/79209)
Supplement: Multimedia Appendix 1 [file formative_v10i1e79209_app1.docx]

Multimedia Appendix 1. Patient scenarios.

Case scenario 1 - Female unexpected weight loss

1. **“**Unexpected weight loss detected: In women aged 60–79, prioritize testing for thyroid function and screening for depression while considering cancer investigation.”
2. Unexpected weight loss detected:
   1. Consider abdominal CT if: persistent mid-thoracic back pain, upper abdominal pain, nausea, or change in bowel habits.
   2. Consider a pancreatic protocol CT scan if: (i) a family history of pancreatic cancer or a genetic mutation that increases risk; (ii) a history of chronic pancreatitis; or (iii) diabetes diagnosed in the previous 6 months.

**1. Patient Information**

- Name: Andrea Smith
- Age: 60
- Gender: Female
- Occupation: Teacher
- Medical History: Andrea has a history of hypertension. She does not smoke and consumes alcohol occasionally.

**2. Chief Complaint**

Andrea Smith presents to your general practice with the chief complaint of persistent abdominal pain. She describes the pain as a dull ache in her upper abdomen, which has been present for the past two weeks. The pain is intermittent, but it has been gradually worsening, prompting her visit.

**3. History of Present Illness**

Andrea explains that the abdominal pain started gradually and is not associated with any specific activities or meals. She denies any recent trauma or injury to the abdomen. Andrea mentions that she hasn't experienced any changes in her bowel habits, such as diarrhea or constipation, and denies any urinary symptoms. She has not noticed any blood in her stool or urine. No nausea

- *(She consulted 8 weeks ago via telehealth because she thought she was told by a friend she looked like she was losing weight.*
- *She had not noticed but consulted anyway as she was feeling a bit stressed.*
- *No physical examination was performed given telehealth app, and she was asked to come back in a month for weight measurement, but she went to visit family overseas so could not come to her app.*

**4. Review of Systems**

- Constitutional: Abdominal pain
- Gastrointestinal: No nausea, vomiting, or changes in bowel habits
- Urinary: No urinary frequency, urgency, or hematuria
- Respiratory: No cough, shortness of breath, or chest pain
- Cardiovascular: Hypertension, no palpitations or chest pain
- Musculoskeletal: Osteoporosis, no joint pain or muscle weakness

**5. Social History**

Andrea is a teacher and lives with her partner. She has one adult son. She maintains a balanced diet and stays active with daily walks. She does not smoke and consumes alcohol occasionally on social occasions.

**6. Family History**

There is no significant family history of gastrointestinal disorders or cancers.

**7. Medications**

Hypertension: Lisinopril 10 mg daily

**8. Physical Examination**

On examination, Andrea appears comfortable but slightly distressed due to the abdominal pain. Her vital signs, including blood pressure, heart rate, and temperature, are within normal limits. Abdominal examination reveals mild tenderness in the upper abdomen, without palpable masses or organomegaly. There is no rebound tenderness or guarding. Normal PR.

Case scenario 2 - Female new-onset diabetes

Recommendations to follow up at increased risk of cancer

New Type 2 Diabetes Diagnosis:

1. Consider a pancreatic protocol CT scan if increased risk or inadequate response to medication

2. Consider abdominal CT if relevant symptoms

**1. Patient Information**

- Name: Beti Thompson
- Age: 60
- Gender: Female
- Occupation: Lawyer

**2. Medical History**

Beti was diagnosed 6 months ago with type 2 diabetes, and hypertension. She is a non-smoker and does not consume alcohol. She had a hysterectomy 10 years ago due to uterine fibroids.

**3. Chief Complaint**

Beti came back for a check-up. She is taking Metformin 1000 twice a day (she has been very consistent with her medication). She is eating more fruits and veg. Has not managed to exercise more. She had her HbA1c taken 2 months ago but had been too busy to come back.

**3. Review of Systems**

- Constitutional: She seems a bit tired
- Gastrointestinal: no nausea or vomiting
- Respiratory: No cough, shortness of breath, or chest pain
- Cardiovascular: Hypertension, no palpitations or chest pain
- Hematologic: No easy bruising or bleeding
- Endocrine: No heat or cold intolerance, no excessive thirst or hunger
- Neurologic: No headaches, dizziness, or neurological symptoms
- Musculoskeletal: Osteoarthritis, no joint pain or muscle weakness

**4. Social History**

Beti is single and lives alone. She maintains a well-balanced diet and engages in light exercise like walking. She does not smoke or consume alcohol. She feels a bit lonely and stressed about her current situation.

**5. Family History**

Father had pancreatic cancer. Died at 74.

**6. Medications**

Diabetes: Metformin 1000 mg twice daily

Hypertension: Lisinopril 20 mg daily

**7. Lab test results**

Diagnostic HbA1c: 9%

Current HbA1c: 9%

**8. Physical Examination:**

On examination, Beti appears mildly fatigued. Her vital signs, including blood pressure, heart rate, and temperature, are within normal limits. There is no lymphadenopathy or hepatosplenomegaly. Abdominal examination reveals mild tenderness in the epigastric region, but no masses are palpable. Chest examination is unremarkable.

**Case scenario 3 – Male unexpected weight loss**

1. “Unexpected weight loss detected: In men aged 60–79, prioritize cancer investigation and testing for diabetes and chronic obstructive pulmonary disorder (especially in smokers).
2. ”Unexpected weight loss detected: Consider abdominal CT if: persistent mid-thoracic back pain, upper abdominal pain, nausea, or change in bowel habits. Consider a pancreatic protocol CT scan if: (i) a family history of pancreatic cancer or a genetic mutation that increases risk; (ii) a history of chronic pancreatitis; or (iii) diabetes diagnosed in the previous 6 months.

**1. Patient Information**

- Name: Alan Walker
- Age: 72
- Gender: Male
- Occupation: Retired Office Manager
- Medical History: No significant medical history

**2. Chief complaint**

Concerns about abdominal discomfort. He refers to two weeks of bloating and a dull ache in his belly. The pain is 3-4/10 and it does not spread

*(He consulted 8 weeks ago because he thought he was losing weight. He had a decreased appetite but was not fatigued or had a low mood. His physical examination was normal, and he was asked to come back in a month for another weight measurement, but he forgot. His last weight was 2 kilos more than the actor's weight.)*

**3. Review of Systems**

- Constitutional: Weight loss, fatigue
- Gastrointestinal: Abdominal discomfort.
- Respiratory: No cough, shortness of breath, or chest pain
- Cardiovascular: Hypertension, no palpitations or chest pain
- Hematologic: No easy bruising or bleeding
- Endocrine: No heat or cold intolerance, no excessive thirst or hunger
- Neurologic: No headaches, dizziness, or neurological symptoms

**4. Social history**

Alan is married and lives with his wife and two adult children. He reports a balanced diet, no exercise, and no history of substance abuse. He drinks alcohol occasionally on social occasions but does not smoke. He refers to no big stressors and his mood is not depressed.

**5. Family history**

Dad with diabetes

Mum hypertension

No cancer

He has not been tested for diabetes

**6. Physical examination**

Normal.

Case scenario 4 - Male new-onset diabetes.

Recommendations to follow up at increased risk of cancer

New Type 2 Diabetes Diagnosis:

1. Consider a pancreatic protocol CT scan if increased risk

2. Consider abdominal CT if relevant symptoms

3. Check adherence to medication at 3 months

**1. Patient Information**

- Name: Sam Johnson
- Age: 70
- Gender: Male
- Occupation: Accountant

**2. Medical History**

Sam has a history of hypertension for which he takes medication. He is a non-smoker and consumes alcohol occasionally. He has not had any significant medical issues in the past.

**3. Chief Complaint**

Sam was diagnosed with type 2 diabetes 1 month ago and came back for a check-up. He was put on Metformin 500 mg orally once a day. He is eating more fruits and vegetables. Has not managed to exercise more.

**4. Review of Systems**

- Constitutional: Normal
- Gastrointestinal: Normal
- Respiratory: No cough, shortness of breath, or chest pain
- Cardiovascular: Hypertension, no palpitations or chest pain
- Hematologic: No easy bruising or bleeding
- Endocrine: No heat or cold intolerance, no excessive thirst or hunger
- Neurologic: No headaches, dizziness, or neurological symptoms
- Musculoskeletal: No joint pain or muscle weakness

**5. Social History**

Sam is married and lives with his wife and has 3 adult children. He reports a balanced diet, regular exercise (although he feels less energetic recently), and no history of substance abuse. He drinks alcohol occasionally on social occasions.

**6. Family History**

Peter's father died of pancreatic cancer at age 75 **(do not provide this information unless asked**), and his mother has hypertension.

**7. Medications**

Hypertension: Lisinopril 10 mg daily

Metformin 500mg once daily

**8. Lab test results**

One month ago: HbA1c: 8%

No follow-up test.

**9. Physical Examination**

Normal.
