# Supplementary material for: Clinical Decision Support Tool for Early Pancreatic Cancer Detection in Primary Care: Simulation Study
Source: JMIR Form Res. 2026 Feb 6;10:e79209. doi: 10.2196/79209 (PMC12924040; doi:10.2196/79209)
Supplement: Multimedia Appendix 2 [file formative_v10i1e79209_app2.docx]

Multimedia Appendix 2. Interview schedule

**Semistructured interview guide:**

Interviews with GP

**Introductions**

Thank you for agreeing to be interviewed for our study. This interview aims to explore your perceptions of a computer program called Future Health Today, which we are hoping to implement in general practice. This program was designed to aid GPs in identifying people who might benefit from further follow-up or investigations, based on information in their medical record, such as recent weight loss that may be unplanned or unexpected and new onset diabetes that may be at risk of pancreatic cancer.

As a GP, you are the main user of this technology. We would like to hear about your insights on how this technology could affect the consultation and how useful it would be as an aid to flag patients with potential symptoms of concern, even if they are not the patient’s primary concern or reason for their visit.

Before we start, I’d like to assure you that your participation is voluntary, you do not have to answer any questions you do not want to, and you can stop the interview at any point. Also, I’d like to remind you that we are recording the session but that the recording will be stored securely and only used by the researchers. Do you have any questions?

Are you ok to start?

**Opening questions:** To start off with, I’d like to know a bit about you and your professional experience. Can you tell me a bit about yourself?

1. Age
2. Gender
3. How long have you practiced as a GP?
4. Where is your practice located?

Now, I’d now like to talk to you about Future Health Today. Future Health Today was built by the University of Melbourne to help GPs and PNs improve the management of their patients’ health conditions. As you have seen, it is a computer program that identifies patients who might be at risk of a health condition. It also provides links to guidelines that GPs and PNs can use to provide evidence-based care. In this case, it identifies people that may be at risk of undiagnosed pancreatic cancer and in need of further follow-up. You have been given the opportunity to use this tool in consultation.

***(We intend to use some of the dimensions of the sociotechnical model by Sittig and Singh to inform this interview schedule. In particular: Clinical content, Human-computer interface, people, workflow, and communication. Also, Sekhon’s acceptability of the health interventions framework measures affective attitude, burden, perceived effectiveness, ethicality, intervention coherence, opportunity costs, and self-efficacy).***

**Questions about acceptability (technology in general)**

1. Can you tell me what you understand FHT to be? *(intervention coherence)*
2. Can you tell me what you think about the use of this program? (Affective attitude)
3. What did you like the most about the tool? (affective attitude)
4. What did you like the least about the tool?
5. Do you have any concerns about using a program like this in general practice? (ethicality)

**Questions relating to the pancreatic cancer recommendations:**

1. What were your thoughts regarding the use of FHT in consultation?

Potential probing questions:

- 1. If you used it: how did the consultation, go? If not, why did you not use it?
  2. How did it affect the workflow? *(Burden, workflow)*
  3. Were you able to explain to the patient the reason for ordering additional tests or investigations? *(communication)*
  4. Would you like additional clinical and/or patient-facing resources to help explain why additional tests are being ordered?

1. What are your initial thoughts regarding the pancreatic cancer recommendations?

*Potential probing questions:*

- *How clear are they? (Content)*
- *Did you find any of the recommendations surprising?*
- *Did you need more information to understand any of the recommendations?*
- *How confident were you in your ability to use and understand the FHT recommendations? Is there anything that would make you feel more comfortable? (Self-efficacy)*

Regarding the content: We are identifying patients at risk of pancreatic cancer in two different scenarios:

- 1. UWL with risk factors or symptoms
  2. New onset diabetes with risk factors, symptoms or inadequate glycemic management after at least three months of medication.

1. How appropriate did you find the recommendations?
2. What would you think if we flagged every new onset diabetes patient to remind you to look for symptoms or risk factors?
3. How useful do you find the recommendations?

*Potential probing questions:*

- *In the context of your everyday practice, how useful do you think the recommendations are for identifying people at risk of pancreatic cancer? (Burden)*
- *How will/have these influence/d your referral thresholds or test ordering?*
- *Have the cancer recommendations influenced/changed your understanding and management of patients at risk of an undiagnosed pancreatic cancer? (if yes, how? In what ways?)*
- *Were you able to use/see the links attached to the recommendations for more information? Are they useful?*
