# Supplementary material for: Clinical Decision Support Tool for Early Pancreatic Cancer Detection in Primary Care: Simulation Study
Source: JMIR Form Res. 2026 Feb 6;10:e79209. doi: 10.2196/79209 (PMC12924040; doi:10.2196/79209)
Supplement: Multimedia Appendix 3 [file formative_v10i1e79209_app3.docx]

Multimedia Appendix 3

Table S1. Description of framework dimensions.

| **Framework** | **Dimension/Theme** | **Description** |
| --- | --- | --- |
| **Acceptability for health interventions (Sekhon et al)** | Affective attitude | How an individual feels about the intervention. |
|  | Burden | The perceived amount of effort that is required to participate in the intervention. |
|  | Ethicality | The extent to which the intervention has good fit with an individual’s value system. |
|  | Intervention coherence | The extent to which the participant understands the intervention and how it works. |
|  | Opportunity costs | The extent to which benefits, profits or values must be given up engaging in the intervention. |
|  | Perceived effectiveness | The extent to which the intervention is perceived as likely to achieve its purpose. |
|  | Self-efficacy | The participant’s confidence that they can perform the behaviour(s) required to participate in the intervention. |
| **Sociotechnical model (Sittig and Singh)** | Clinical Content | The text, numerical data, images. And computable knowledge that constitute the language of clinical applications |
|  | External Rules | Federal or state rules that facilitate or constrain internal organizations features |
|  | Internal Rules | Policies, procedures, work environment, and organizational culture |
|  | Hardware and software infrastructure | The computing infrastructure used to power, support, and operate=e clinical applications and devices. |
|  | Human computer interaction | All aspects of the computer that users can see, touch, or hear as they interact with it. An interface enables unrelated entities to interact with the system and includes aspects of the system that users can see, touch, or hear. |
|  | People | Everyone who interacts in some way with the system, from developer to end-user, including potential patient-users. This dimension represents the people involved in all aspects of the design, development, implementation, and use of HIT. It also includes the ways that systems help users think and make them feel. |
|  | Workflow and communication | Processes to ensure that patient care is carried out effectively. |
|  | System measuring and monitoring | Processes to evaluate both intended and unintended consequences of health information technology implementation and use. |

**References**

1. Sekhon M, Cartwright M, Francis JJ. Acceptability of healthcare interventions: an overview of reviews and development of a theoretical framework. BMC health services research. 2017 Dec;17:1-3.
2. Singh H, Sittig DF. A sociotechnical framework for safety-related electronic health record research reporting: the SAFER reporting framework. Annals of Internal Medicine. 2020 Jun 2;172(11_Supplement):S92-1
