# Supplementary material for: Clinical Decision Support Tool for Early Pancreatic Cancer Detection in Primary Care: Simulation Study
Source: JMIR Form Res. 2026 Feb 6;10:e79209. doi: 10.2196/79209 (PMC12924040; doi:10.2196/79209)
Supplement: Multimedia Appendix 4 [file formative_v10i1e79209_app4.pdf]

Multimedia Appendix Table:

Coreq Checklist. Clinical Decision Support Tool for Pancreatic Cancer in Primary Care: A Simulation Study

			Reported?	Page	What? How is it portrayed in the text
**Domain 1: Research team and reflexivity**					
Personal Characteristics					
1.	Interviewer/facilitator	Which author/s conducted the interview or focus group?	Yes	10	2. Coding Process: JMG and KS independently coded the transcribed interviews using NVivo 14. We systematically categorised each code under the relevant themes derived from our theoretical frameworks, ensuring a consistent application of the frameworks' components.
2.	Credentials	What were the researcher's credentials? *E.g. PhD, MD*	Yes	11	JMG is a trained GP and PhD candidate at the Department of General Practice and Primary Care, University of Melbourne. KS, a researcher in the Department, holds an MPhil and has been actively involved in primary care research since completing postgraduate studies
3.	Occupation	What was their occupation at the time of the study?	Yes	11	JMG is a trained GP and PhD candidate at the Department of General Practice and Primary Care, University of Melbourne. KS, a researcher in the Department, holds an MPhil and has been actively involved in primary care research since completing postgraduate studies.
4.	Gender	Was the researcher male or female?	Yes	11	They are both females
5.	Experience and training	What experience or training did the researcher have?	Yes	11	They are both females, experienced in qualitative research, and together they bring a complementary blend of clinical expertise and research experience to the study.

Relationship with participants					
6.	Relationship established	Was a relationship established prior to study commencement?	Yes	11	Neither had an established relationship with study participants.
7.	Participant knowledge of the interviewer	What did the participants know about the researcher? e.*g. personal goals, reasons for doing the research*	Yes	11	“Neither had an established relationship with study participants” and “All participants received a plain language statement (PLS) explaining the study and its objectives. No other information was provided regarding personal goals or reasons for doing the research”
8.	Interviewer characteristics	What characteristics were reported about the interviewer/facilitator? e.g. *Bias, assumptions, reasons and interests in the research topic*	Yes	11	No other information was provided regarding personal goals or reasons for doing the research.
**Domain 2: study design**					
Theoretical framework					
9.	Methodological orientation and Theory	What methodological orientation was stated to underpin the study? *e.g. grounded theory, discourse analysis, ethnography, phenomenology, content analysis*	Yes	10	We employed reflexive thematic analysis, which conceptualises meaning as constructed through the researcher's interpretative process rather than inherent in the data. This approach recognises that new meanings are always theoretically possible, as analysis is a situated, reflexive, and theoretically embedded practice of knowledge generation.
Participant selection					
10.	Sampling	How were participants selected? *e.g. purposive, convenience, consecutive, snowball*	Yes	7	We emailed a convenience sample of 72 GPs from a database available at the Department of General Practice, focusing on those with current connections to the department, as they needed to attend the simulation laboratory, and posted on social media platforms and

					groups associated with the University. We excluded from our invitation GPs who had participated in similar studies to avoid potential bias from their acquired 'expertise' in simulated scenarios. We recruited all GPs who responded to our initial recruitment invitation, distributed via email and social media groups, and were able to attend the simulation laboratory.
11.	Method of approach	How were participants approached? e.*g. face-to-face, telephone, mail, email*	Yes	7	We emailed a convenience sample of 72 GPs from a database available at the Department of General Practice, focusing on those with current connections to the department, as they needed to attend the simulation laboratory, and posted on social media platforms and groups associated with the University. We excluded from our invitation GPs who had participated in similar studies to avoid potential bias from their acquired 'expertise' in simulated scenarios. We recruited all GPs who responded to our initial recruitment invitation, distributed via email and social media groups, and were able to attend the simulation laboratory.
12.	Sample size	How many participants were in the study?	Yes	11	Of the 72 GPs invited, 12 responded and 11 participated (one GP was not available at the time of the simulation sessions).
13.	Non-participation	How many people refused to participate or dropped out? Reasons?	Yes	11	Of the 72 GPs invited, 12 responded and 11 participated (one GP was not available at the time of the simulation sessions).
Setting					
14.	Setting of data collection	Where was the data collected? e.*g. home, clinic, workplace*	Yes	8	Following completion of the simulated consultations, the GPs participated in a single semi-structured interview on site (Multimedia Appendix: Interview Schedule), assessing

					the acceptability, feasibility of the CDSS and its impact on workflow.
15.	Presence of non-participants	Was anyone else present besides the participants and researchers?	Yes	8	There were no external people to the study present during the simulation or the interviews
16.	Description of sample	What are the important characteristics of the sample? *e.g. demographic data, date*	Yes	11	Approximately half of the GPs encountered female patient scenarios. The demographic characteristics of the participants are described in Table 1. Seven GPs were aged in their thirties and had fewer than 10 years in practice, eight were female, and seven were Australian born. Most GPs practised in metropolitan practices, one had experience in rural practices, and two worked in an Aboriginal Medical Service.
Data collection					
17.	Interview guide	Were questions, prompts, guides provided by the authors? Was it pilot tested?	Yes	8	The interview guides were developed using relevant dimensions of two published frameworks:<br>1. Sociotechnical model for evaluation of digital interventions by Sittig and Singh [50,51].<br>2. Sekhon's acceptability of healthcare interventions [52].
18.	Repeat interviews	Were repeat interviews carried out? If yes, how many?	Yes	10	no repeat interviews were conducted.
19.	Audio/visual recording	Did the research use audio or visual recording to collect the data?	Yes	8	Each session took 10-15 minutes, consistent with consultation times in Australian general practice, and was observed, filmed and audio recorded by the researchers through a one-way mirror
20.	Field notes	Were field notes made during and/or after the interview or focus group?	Yes	10	Field notes were not developed during or after the interviews

21.	Duration	What was the duration of the interviews or focus group?	Yes	9	Interviews lasted approximately 30-45 minutes
22.	Data saturation	Was data saturation discussed?	Yes	10	We employed reflexive thematic analysis, which conceptualises meaning as constructed through the researcher's interpretative process rather than inherent in the data. This approach recognises that new meanings are always theoretically possible, as analysis is a situated, reflexive, and theoretically embedded practice of knowledge generation. Consequently, the concept of data saturation, which assumes a point where no new information emerges, was not suited to our study. Instead, our sampling strategy focused on recruiting participants who could provide rich, relevant information to address our research questions. This approach ensured we captured a range of perspectives from those willing and able to participate, rather than aiming for a predetermined point of "informational redundancy" [53].
23.	Transcripts returned	Were transcripts returned to participants for comment and/or correction?	Yes	10	Interview participants were not asked to provide feedback on the transcripts, nor the findings and no repeat interviews were conducted.
**Domain 3: analysis and findings**					
Data analysis					
24.	Number of data coders	How many data coders coded the data?	Yes	10	2. Coding Process: JMG and KS independently coded the transcribed interviews using NVivo 14. We systematically categorised each code under the relevant themes derived from our theoretical frameworks, ensuring a consistent application of the frameworks' components.

25.	Description of the coding tree	Did authors provide a description of the coding tree?	No		We provide a description of the main themes in Multimedia Appendix Table: Description of Framework Dimensions instead
26.	Derivation of themes	Were themes identified in advance or derived from the data?	Yes	10	1. Framework Integration: Each dimension from both frameworks was used as an overarching theme in our codebook. This approach ensured a comprehensive and structured analysis aligned with established theoretical concepts.
27.	Software	What software, if applicable, was used to manage the data?	Yes	10	2. Coding Process: JMG and KS independently coded the transcribed interviews using NVivo 14. We systematically categorised each code under the relevant themes derived from our theoretical frameworks, ensuring a consistent application of the frameworks' components.
28.	Participant checking	Did participants provide feedback on the findings?	Yes	10	Interview participants were not asked to provide feedback on the transcripts nor the findings
Reporting					
29.	Quotations presented	Were participant quotations presented to illustrate the themes / findings? Was each quotation identified? e.*g. participant number*	Yes	11-16	Results<br>Eg: “So, it's an interesting tool. But it'll just augment your thinking, and just remind you of things, which is always useful when you're busy and tired.” GP9
30.	Data and findings consistent	Was there consistency between the data presented and the findings?	Yes	11-16	Eg; Collectively, the GPs valued the gentle language of the prompts and resources provided by the prompts for further reading.<br>“I think it's very clear, very simple. There's no ambiguity just says it's this patient's got new type two diabetes diagnosis. So, consider looking for pancreatic cancer. It’s really simple.”GP1

31.	Clarity of major themes	Were major themes clearly presented in the findings?	Yes	11-16	Table 2 and text
32.	Clarity of minor themes	Is there a description of diverse cases or discussion of minor themes?	Yes	11-16 and appen dix	Text and Multimedia Appendix Table: GP thought process<br>Eg: While they collectively agreed that the point-of-care flags served as gentle reminders, some GPs also expressed concerns that it might prompt them to focus solely on pancreatic cancer, potentially overlooking other possible diagnoses, for patients with unintended weight loss and new-onset diabetes.<br>“But it did kind of push me towards focusing on that. And is that a good thing or not? Well, I guess it raises the question of in someone who's just with diabetes, who's tired, what's the probability they have pancreatic versus some other cause? And even though I guess it's really important to know about pancreatic cancer, would it make me forget about other diagnoses.” GP2

Tong A, Sainsbury P, Craig J. Consolidated criteria for reporting qualitative research (COREQ): a 32-item checklist for interviews and focus groups. International journal for quality in health care. 2007 Dec 1;19(6):349-57.
