# Supplementary material for: Clinical Decision Support Tool for Early Pancreatic Cancer Detection in Primary Care: Simulation Study
Source: JMIR Form Res. 2026 Feb 6;10:e79209. doi: 10.2196/79209 (PMC12924040; doi:10.2196/79209)
Supplement: Multimedia Appendix 5 [file formative_v10i1e79209_app5.docx]

Multimedia Appendix 5.

Table S1. Thought process of decision-making for the GPs.

This theme directly captures the clinicians' perspectives on the Pancreatic Cancer recommendations. The key feedback from each GP is summarized in the table below:

| **Key feedback** |
| --- |
| **GP1** |
| Reflecting on effectively communicating the need for a CT scan to patients, it is important to justify that the recommendation is based on medical evidence and research, given the time and cost involved. GPs should schedule longer consultations when discussing the possibility of a CT scan. GP1 repeatedly mentioned the importance of having strong reference materials to support the recommendation and also emphasized the importance of involving the patient in the decision-making process.  *“If I reflect on my own practices, when I've had patients come in for on for nonspecific presentations, managing uncertainty takes up a lot of the bulk of the consultation, just you sort of thinking about all the different possibilities where to go next. Sometimes there's a financial aspect to look for, like not all patients can access private care. The other thing to consider sometimes like rebated investigations, it's not .. CT Abdo, but MRI differently in some ultrasound. I think with this kind of consultation, having a way of explaining the evidence basis for why you're making those decisions can be good. I think a patient would probably appreciate knowing that that you're not just coming up with this randomly, you're coming up with this because somewhere in the background, someone's done work that's shown these associations. And so we're looking at the case with good practice in mind and evidence based practice, which I think is really important.”* |
| **GP2** |
| Being a GP who is interested in pancreatic cancer with clinical experience on picking up patients with the condition, wonders if it is good being pushed to focus on pancreatic cancer but loved that the POC prompted to do more reading on the provided references. Similar to GP1, GP2 indicated on patient circumstances given the CT scan cost and time.  *“Well, clearly in the first patient, you know, weight loss, it was kind of very good to have a reminder, but I think I probably would have done something anyway, with the second patient. I guess it made me think of there's something here, I'd better dig a bit deeper. Whereas normally, I guess if I saw someone who was more tired, and I tried to be true to what I would normally do, which is check that he's not depressed and check, there's nothing else going on. And I guess in the real world, if I hadn't had that prompt, I would have just done some Bloods and given him some lifestyle advice and seen him again in a couple of weeks. And then I might have thought later about doing some more screening for cancers. But it did kind of push me towards focusing on that. And is that a good thing or not? Well, I guess it raises the question of in someone who's just with diabetes, who's tired, what's the probability they have pancreatic versus some other cause? And even though I guess it's really important to know about pancreatic cancer, would it make me forget about other diagnoses? And that's where I'm not sure about that. But it's prompted me to think and it's prompted me to do more reading. So in that regard, it's, it's good… Am I going to wait for him to get symptoms, and here, it definitely prompted me to say, well, he's got all those four boxes, and not that I better move ahead with a scan. But I think if I hadn't read that, I would have said, let me do the blood tests first. And if the blood tests are completely normal, I might have given him some something else thinking what else would I have done? … I wouldn't have thought of pancreatic cancer as a first there. I'm sure it happens. But I don't think it would happen often.*  *But again, that's, again, if this guideline said any unexplained bloating, goes straight to pelvic CT, I probably would follow what the future health today told me as long as the patient was happy with it. And I guess you will say you making all these other decisions aren't cheap.”* |
| **GP3** |
| Well received the prompts. Similar to earlier GPs, GP3 mentioned about timing of consult. Prioritized and concerned on communicating to the patient based on recommendation and without alarming the patient.  *I think it's really good. Because there's a lot to try and, you know, be across and think about, and particularly for important things, I think, like, you know, cancer screening or, you know, possible signs of cancer, I think it is a really good reminder, you know, obviously, you're trying to be integrating that with everything else. And, you know, I guess Yeah, some, you know, as you know, like, sometimes the patients are coming in with, and they might mention the weight loss, but it's one of hundreds of different things and, you know, social chaos and like what's going on? So, you know, I guess it's always gonna depend on, you know, the flow of that consult, and, you know, as to how much you can look and kind of use those recommendations at that point in time. But I think, yeah, you know, it's one can, seems to be like it can feeling well as one thing you can integrate into your decision making. Yeah, I mean, I think in those particular consults, it did. Yeah, it did prompt me to think of things that I wouldn't necessarily straightaway think of. So that was good.*  *... I mean, the second one, it would never to be honest. Like, I wouldn't think about , you know, new diagnosis, diabetes, obviously. So common, I wouldn't necessarily, I wouldn't really probably think about oh, I need to ask her about symptoms of pancreatic cancer, like it just wouldn't probably occur to me.*  *Yeah, it's hard. I don't know, I've still obviously, only my clinical communication, like because you want to sort of you want the patient to know that it's important. ... I think we really need to do this because it could be something serious. But how do you communicate that without making them panic?* |
| **GP4** |
| Similar to GP3, GP4 mentioned that for the second scenario, wouldn’t have straight away thought of pancreatic cancer. GP4 also stressed that she would require HbA1c profile to compare and consider the recommendation. Again, they mentioned reconsidering the recommendation given the test is costly and time consuming. Similar to GP2, GP4 mentioned the recommendation is making a GP focus on PC but what about other possible conditions.  *I mean, maybe this is my bias, but I would assume if someone had a family history of pancreatic cancer, or genetic mutations, that's usually something that they would offer. And certainly with like new diagnosis of diabetes, I'm certainly aware it's a thing that it can be related to pancreatic cancer, but it's not something that I would generally think about. Like if the HbA1c been like, 14, or if we repeated it, and it had gone up, then definitely I would be thinking about it that I think to start from the single HbA1c I wouldn't be .., I don't know, maybe that's wrong.*  *I mean, I guess the thing is, I know pancreatic cancer is under like, it's often a delayed diagnosis. But it's still pretty uncommon. And what is really common is diabetes. And it's often not managed, particularly well. So I guess in terms of my priorities, it's always going to be trying to do the basic diabetes management stuff that isn't done well. Rather than getting sidetracked about pancreatic cancer risk, I think realistically, unless there was something that really seemed really off, like the HbA1c had jumped up, or it was like really high a diagnosis, or that type of thing.*  *Like, I don't know, to me, I like to have some sort of baseline something before sending people off to potentially expensive and time consuming tech.*  *And then I guess the other thing I was thinking of is like, Oh, we're focused on like, because this is a pancreatic cancer ones, we're focused on CT..like, What happens if she just needs to get gastroscopy? But I'm just ignoring that because this is talking about CT. So I had, I guess I had that in mind* |
| **GP5** |
| Another GP with real clinical experience of PC, would hesitate to action the recommendation until further investigation is done on the patient. Stressed about the importance of HbA1c profiling for the new-onset of diabetes to better understand if it was a gradual increase in the reading or sudden spike to be able to justify their clinical decision in suggesting CT scan. Particularly commented on improving the language of the recommendation to be clearer to guide GP in considering decision making over consultation with regards to PC.  *I had a patient not a couple of years ago, who presented with a sudden diabetes with a HbA1c of nine or 10… HbA1c.. like you're fasting sugar, 18 months ago is normal. What, how, how on earth did this happen? …But she had a fullness in the epigastrium, When I examined her like an obvious fullness. So I sent her straight for a CT, she had a big cancer, and she died within six weeks. So I think that HbA1c quite surprised me and flat and it stuck with me. So then I'm seeing this lady, ... I decided for this patient to leap to a CT, was not required... But enough for me to say, I actually want to see what your bloods are doing. Including your pancreas, Bloods, with the HbA1c of nine and bring you back. If that makes sense.*  *So that third point, so wanting to make sense, .. is that saying, if they're not responding to, to treatment, then do the CT. ... So you might want to actually state that if diabetic control is not responding, or if Diabetes is not responding to treatment, that might be a better statement. Because in this particular patient, we had didn't have any follow up Bloods. But if we had no HbA1c it actually not improved or increased, though, saying that she we have a patient who I'm not I've no idea what a previous glycemic control had been like, and I don't really know what she hadn't seen the diabetes, she hadn't had proper education. So I don't know if what she was doing at home. Was it all going to be useful or not? Just another reason why I didn't do a CT. Like I don't have a good grasp on what's really happening here.* |
| **GP6** |
| Hesitates on actioning the recommendation for CT scan as not familiar with the guidelines and would like more evidence to implement and prefers prioritizing diabetes management for new-onset diabetes. (closely aligned with GP4, GP10, GP7)  *I think consider a CT scan leads me to where I was before, which is that I wouldn't order, this is a very soft recommendation. So I guess again, that I (need) statistics or something to justify. And, I mean, I like numbers... the fact that based on this flowchart, … I would have thought about ordering, as opposed to immediately discounting a CT scan in the first patient with weight loss and abdominal pain. .. And I didn't ask the first the second patient if she has a family history of pancreatic cancer, if she did know, I probably wouldn't order. I think, yeah. I think that I don't like to order tests. With a 1% chance of finding something, even if it's significant, I guess, in this vague and differentiated stage, it's not like I'm saying I'd never ordered a CT. But if she just had diabetes, a 60-year-old woman with diabetes, it's not an uncommon occurrence. She happens to have a family history of pancreatic, pancreatic cancer, I probably would see how her diabetes is going. And if it had responded well to medication, I think I'd go it's diabetes. So they've only got to change my management. Even if I had asked about the family history of pancreatic cancer, I think I still would have said, Do your bloods see you and see you with results? And we'll see how you respond to medication…* *I understand the recommendation, I just .. I don't agree with it.* |
| **GP7** |
| Similar to GP5 & GP6, GP7 would prioritize diabetes management compared to PC as well as considers consultation timing as important prior to communicating about PC to patient.  *I think the idea of clinical decision support tools is awesome. And I think definitely useful. This one I found a little bit hard, because it's looking for pancreatic cancer, and it's a CT and the investigation… because I was already looking at it being like, oh, gosh, that's like a big thing for a patient with a new diagnosis of diabetes, too. You don't want to worry them further about, oh, you might have pancreatic cancer, but you need to find a way to use that tool without freaking them out. And definitely, if you like you've used a tool before, then you're more likely to integrate the questions into your history. So that would be a bit easier. But I think it's also probably going to be about timing as to when you use that, like new diagnosis of diabetes. While that is probably an ideal time to then go looking for pancreatic cancer. Also seems like maybe not as ideal a time because the patient is also reeling from her diagnosis of diabetes, and how to manage all of that. Yeah. And we're only doing like, you know, stepwise management at the moment anyway, like, there's so many things we have to do in terms of diabetes, and I don't want to add on an abdominal CT scan.* |
| **GP8** |
| After trying actioning the recommendation during the simulation sessions, a junior GP indicated that communicating to patient about PC was a major factor (similar to earlier GPs) and highlighting using time as a tool or strategy towards slowly actioning the recommendation if needed.  *Like this is written in medical language for the clinicians to read, and you have to do like some kind of translation between this and then what comes out of your mouth to the patient. And I think that takes practice…*  *I think a main, mind communication, clunky I felt like I had to backpedal and be like, oh, sorry. So how do you feel about all of this? After the patient reacted in a way that made me think, oh, gosh, I've just totally freaked her out. So yeah, it had a negative impact on my communication today, I would say for sure…That, you really do need to make the time to explain things properly, I think. Yeah. So yeah, definitely a risk. The communication for sure. It's a complex like vague, cancer symptoms are complex, and it's hard to convey that to patients quickly, in a way that doesn't make them panic. But that also keeps them informed of what's happening because the worst set scenario is that they go back they can't get their scan and they've got cancer and you're breaking bad news and they had no idea it was coming. So yeah, it is tricky..*  *And also as a new young GP, I'm getting more comfortable watching and waiting and saying to the patient come back in a week or come back in two weeks. And then using the time has a tool to decide whether or not this needs to be investigated further. So if she came back in one or two weeks, and the pain is getting worse, and all of a sudden, I have no appetite, and I've been getting sweats, and I'm losing weight or all these things and okay, like 100% I'm gonna do these scans.* |
| **GP9** |
| Commented that the use of the FHT-tool might get smoother with regular usage and a good reminder as well a safety net in a busy practice routine.  *Yeah,.. it's interesting… And obviously, you know, it's the first time I've used it, and things become more smooth as you use it more and more…Yeah, it was good. I noticed it. So, before he came in main thing the first time I saw it. I was focused on a bit…Oh, that's interesting. I should think keep that in the back of my mind. You do the general history, generally exam, general workup. And I think when you're sitting at the near the end of the consult of your diagnostic process, and you ask yourself that question of what am I missing? Have I missed anything here? At least that's what I always do. When it's a bit unclear, like fatigue, you always sit there and go what could this be that I'm ever looking that's going to kill someone? That's a good little prompt, you know, or it could be like there might be a feature of pancreatic cancer risk, we thought about Thalassemia or whatever. Now talk to him about it. In my mind, I weighed up whether or not I was relying too much on it. Was it swaying me?*  *So it's an interesting tool. But it'll just augment your thinking, and just remind you of things, which is always useful when you're busy and tired.* |
| **GP10** |
| Being an experienced GP and cancer expert, GP10 feels that the recommendation is not appropriate for discussion with new-onset diabetes patient and stressed that diabetes management should be prioritized before cancer risk discussion.  *And I'm not putting up my GP but as an expert in cancer. 70. Okay. Has he had the talk about PSA testing informed consent? Has he had the national bowel cancer screening given 30%? Good 70% at home on a bookshelf. Okay, well, skin cancer, okay, now because of diabetes. Okay, we're going to think pancreatic cancer, if he doesn't smoke, okay, no need to think of lung but maybe with...FHT doing some recommendations that I guess even space, but (is it) practical to the GP? Where once you've got a chronic disease stable with diabetes, or let's say blood pressure, no symptoms, then you say at 12 months, could you consider a care plan, but also a risk identification for all cancers?*  *…And I'm just going through the mind is that if let's say this pops up, and the fact that consider protocol can see abdomen if relevant symptoms, this is the cue where it comes up. But at six to 12 months, this is where we can have that more nuanced conversation, because I don't think we can do in the first three months.* |
| **GP11** |
| Similar to other GPs, GP11 expressed concern about using software like FHT has potential bias focusing on targeted diagnosis eg: PC and missing on other possible conditions. He also mentioned that the prompt reduced threshold for CT scan. Given his experience working in various settings, he reconsiders actioning the recommendation based on patient circumstances from rural or urban settings.  *I think once you see it, then you see what it's doing. I can understand why it's there that you've chosen to install this add on, I suppose to the to the EMR. Yeah, so I think it would be important to clarify with whoever's using it, or you use this only for pancreatic cancer. And if you only gonna see it if if it's flagging someone as a high risk of pancreatic cancer. But I guarantee then the next question will be well, what about everything? Everything else?*  *Yes, I think I think if it was, in reality, I think you've probably reduced the threshold of me wanting to do a scan. Because it puts that in your head live on the fly. And the barriers, I guess, again, it depends on where you are physically in the country, and the access to the testing facilities and the costs and all of that. So when I was working in a rural place, I would think, three or four times before referring someone for a scan, because it was a very laborious task for the patient. And there was limited availability. But if you're in suburban Melbourne, where you can trip over a radiology practice, in every corner, you might end up with definitely overdoing things in a population that might not need it. I don't know if I would, again, when you're seeing it for the first time, you might overdo it initially, and then it might taper off over time.* |
